# Bibliographical Notices

**Published:** 1855-06

**Authors:** 


*An Outline of Medical Chemistry, for the Use of Students.* By B. Howard Rand, A.M., M.D., Professor of Chemistry in the Philadelphia College of Medicine, etc. Philadelphia: Lindsay & Blakiston. 1855.

This little work is intended as an outline sketch to assist the medical student in acquiring a knowledge of this most necessary science. It contains an extremely laconic abridgement of the usual systematic course, as met with in the text books most in use in our colleges, attention being more particularly paid to those combinations that are used in medicine, and are officinal in our Pharmacopœia, to which work it may form a useful companion in those cases in which the student wishes to obtain a rapid survey of the field of medical chemistry.

---

*The Principles and Practice of Obstetrical Medicine and Surgery, in reference to the process of Parturition, with sixty-four plates and numerous wood-cuts.* By FRANCIS H. RAMSBOTHAM, M. D., &c. A new American edition, revised by the author. With Notes and Additions by WILLIAM V. KEATING, M. D., A. M. &c., Philada. Blanchard & Lea, 1855.

Few reprints from the British press have ever met with more favor from American practitioners than the above work of Dr. Ramsbotham. "Chaste in language, classical in composition, happy in point of arrangement, and abounding in most interesting illustrations," the emphatic language applied to it by Professor Hodge, so well expresses its deserts that we consider it unnecessary to dwell further upon them. The present edition is rendered even more valuable than the previous one, from the additional material published in the interval, so that it may now be considered one of the best summaries of the principles and practice of obstetrics in the English language.

Dr. Ramsbotham devotes a long article, 26 pages, to the history and effects of anæsthesia in labor. "Hitherto," he says, "I fear we have seen too much of the bright side of the picture; the feelings and imagination have been allowed to outstrip the judgment; and the glorious anticipations indulged in have, by their dazzling lustre, unfitted the mind for patient analytical examination. But the period has now arrived, when, from the facts collected, a dispassionate opinion may be formed; when the excitement of the novel, bold and startling idea having passed away, over-zeal may be tempered by discretion, and prejudice corrected by calm, sober, and philosophic reasoning." A discussion of the effects of anæsthetic agents upon the health of both mother and child follows this preamble, which our limits alone prevent us from giving more in detail. The action of chloroform is divided, upon the authority of Dr. Snow, into five stages or degrees. In the 1st, there is a kind of inebriation, which is usually agreeable, consciousness to surrounding objects, remaining, however, unimpaired. In the 2d the mental functions are impaired, but not entirely suspended; consciousness is no longer correct, and a dreamy state, analogous to delirium, supervenes. The patient is not *dead drunk*, but only in the state described by the law as *drunk and incapable.* In the 3d stage, all voluntary motion is paralysed; the mental functions are completely in abeyance. It does not follow, however, that an operation may be commenced immediately the narcotism reaches this degree, for anæsthesia is not a necessary part of it. The fourth degree brings with it relaxation of the voluntary muscles, with complete insensibility to external impressions, so that no pain is felt even on the infliction of severe personal injuries. The breathing is not unfrequently attended with some degree of stertor. The fifth degree is the *commencement of dying.* Dr. R. argues the great danger of chloroform, from the fact that perfect freedom from pain cannot be expected, unless the fourth degree be induced, when sensibility as well as sensation and muscular motion, is completely destroyed; "two or three more sniffs voluntarily allowed and the fatal gulf is crossed." It is also, a cumulative medicine; its effects increasing after its administration has been discontinued. These and the unequal susceptibility of patients to its influence, and its incompatibility with serious disease of the circulatory or respiratory organs, are the principal reasons brought forward against its use. Besides death, which has assuredly, he says, occurred from its administration, many minor casualties may be apprehended from its action. Three instances of puerperal mania have come within his own knowledge, which were induced by it. Paralysis, hysteria, and epileptic fits are also occasionally caused by it. The condition that must exist before a patient can be rendered oblivious to the sufferings incidental to her situation, he says, emphatically, "is not sleep, but drunkenness; the only kind of sleep, if sleep it can be called, in the least degree analogous to it, is that death-like insensibility into which a person is hurled when stupified by spirituous liquors; a state but little removed from apoplexy; and which indeed in very many instances has eventuated in a seizure of that dreadful malady." There are only one or two contingencies in which he can even imagine its influence to be applicable. When the fœtus lies transversely in utero, the membranes having been ruptured for some hours and the patient obstinately refuses to submit to delivery, he should deem it his duty to employ etherization, taking advantage of its effects to perform the operation. The same principle would guide him in some other operative cases where the forceps were required, and their application perseveringly resisted.

Such are the opinions of our author concerning anæsthesia in labor. It is but proper that we should remark that the American editor enters a warm protest against them. As his experience, however, has been entirely confined to *ether*, while Dr. Ramsbotham's arguments refer to chloroform, we shall not discuss his conclusions.

As we stated previously, the work has been considerably enlarged by the author since the last issue. The additions of the American editor, which cannot, both from their number and value, but prove acceptable to the reader, consist in a description of the inclined planes of the pelvis; generation; signs of pregnancy; fœtal circulation; the mechanism of labor; the employment of anæsthetics, to which we have just referred; the use of ergot, &c., and other matters unnoticed by the author. The printing, paper and illustrations deserve the highest commendation.

